# Gut microbiota from B-cell-specific TLR9-deficient NOD mice promote IL-10^+^ Breg cells and protect against T1D

**DOI:** 10.3389/fimmu.2024.1413177

**Published:** 2024-06-06

**Authors:** Xin Yang, Juan Huang, Jian Peng, Pai Wang, F. Susan Wong, Ruirui Wang, Dapeng Wang, Li Wen

**Affiliations:** ^1^ Department of Food Science and Technology, School of Agriculture and Biology, Shanghai Jiao Tong University, Shanghai, China; ^2^ Section of Endocrinology, Internal Medicine, School of Medicine, Yale University, New Haven, CT, United States; ^3^ Department of Gastrocolorectal Surgery, General Surgery Center, The First Hospital of Jilin University, Changchun, Jilin, China; ^4^ Division of Infection and Immunity, Cardiff University School of Medicine, Cardiff, United Kingdom; ^5^ Shanghai Innovation Center of Traditional Chinese Medicine (TCM) Health Service, Shanghai University of Traditional Chinese Medicine, Shanghai, China

**Keywords:** type 1 diabetes, gut microbiota, interleukin-10, gut permeability, immune tolerance, NOD mice, Toll-like receptor 9, B cells

## Abstract

**Introduction:**

Type 1 diabetes (T1D) is an autoimmune disease characterized by the destruction of insulin-producing β cells. Toll-like receptor 9 (TLR9) plays a role in autoimmune diseases, and B cell-specific TLR9 deficiency delays T1D development. Gut microbiota are implicated in T1D, although the relationship is complex. However, the impact of B cell-specific deficiency of TLR9 on intestinal microbiota and the impact of altered intestinal microbiota on the development of T1D are unclear.

**Objectives:**

This study investigated how gut microbiota and the intestinal barrier contribute to T1D development in B cell-specific TLR9-deficient NOD mice. Additionally, this study explored the role of microbiota in immune regulation and T1D onset.

**Methods:**

The study assessed gut permeability, gene expression related to gut barrier integrity, and gut microbiota composition. Antibiotics depleted gut microbiota, and fecal samples were transferred to germ-free mice. The study also examined IL-10 production, Breg cell differentiation, and their impact on T1D development.

**Results:**

B cell-specific TLR9-deficient NOD mice exhibited increased gut permeability and downregulated gut barrier-related gene expression. Antibiotics restored gut permeability, suggesting microbiota influence. Altered microbiota were enriched in Lachnospiraceae, known for mucin degradation. Transferring this microbiota to germ-free mice increased gut permeability and promoted IL-10-expressing Breg cells. Rag^-/-^ mice transplanted with fecal samples from *Tlr9* ^fl/fl^ *Cd19*-Cre^+^ mice showed delayed diabetes onset, indicating microbiota’s impact.

**Conclusion:**

B cell-specific TLR9 deficiency alters gut microbiota, increasing gut permeability and promoting IL-10-expressing Breg cells, which delay T1D. This study uncovers a link between TLR9, gut microbiota, and immune regulation in T1D, with implications for microbiota-targeted T1D therapies.

## Introduction

Type 1 diabetes (T1D) is a chronic autoimmune disease characterized by insulin deficiency and resultant hyperglycaemia (1). T lymphocytes, other subsets of immune cells and molecules of innate immunity play an important role in mediating and modulation of the immunopathogeneisis of T1D development leading to insulin deficiency (2). TLR9, an important innate immune receptor, recognizes guanine-cytosine–rich DNA from pathogens and self-DNA as well as short single-stranded synthetic DNA 5′-cytosine-phosphate-guanine-3′ (CpG) (3). TLR9 plays an important role in the development of some autoimmune diseases (4), which include systemic lupus erythematosus (SLE), autoimmune thyroiditis (5) and autoimmune nephropathy (6). Our previous work found that the incidence of autoimmune diabetes was significantly delayed in systemic TLR9 deficient and in B cell-specific TLR9-deficient NOD mice (7, 8). This protection was partly mediated by increased expression of immunoregulatory interleukin-10 (IL-10) (8), enhanced expression and regulatory function of CD73^+^ T cells as well as improved islet β-cell function (7, 9).

In addition to genetic factors, the rapid increase in the incidence of T1D in the past three decades indicates that environmental factors may play an important role in T1D development (10). Gut microbiota, as one of the critical environmental factors, may work as a mediator in the development of T1D and this hypothesis has been supported by the studies in both animal models and human studies (11–14). The gut microbiota influence the development of T1D through multiple modalities, one of which is altered intestinal barrier function as a result of the dysbiosis of gut microbiome that appears to contribute to T1D development (15). However, the current knowledge regarding the role of intestinal barrier in T1D development is inconsistent. Some studies have shown that the change of gut permeability due to the altered microbiota and their metabolites contributed to the development of T1D in animal models through recruitment of islet-reactive T cells (16), increased permeability to luminal antigens (17) and amplified immune signaling cascades (18). However, low-dose chemicals induce insulin-dependent diabetes in mice without affecting intestinal permeability, indicating that the increased intestinal permeability is not absolutely required for the development of T1D in this animal model (19). This notion is also supported by the absence of differences in intestinal permeability in diabetic NOD vs age-matched non-diabetic NOD mice (20). Indeed, therapies aimed at improving the intestinal barrier alone have little effect on altering T1D development (20, 21). It is clear that further investigation into the relationship between intestinal permeability, which is influenced by multiple factors, and the development of T1D is needed.

Trillions of microorganisms co-exist symbiotically with their hosts, making essential contributions to the host metabolism and immune system (22). Results from experiments with animals and clinical trials in humans have shown that after fecal microbiota transplantation, the gut microbiota shifted to a metabolic phenotype similar to that of the fecal donors (23–25). There are a large number of B cells among the immune cells in the mucosal associated lymphoid tissues (MALT) and these express high levels of TLR9. B cells produce anti-microbial antibodies that are highly present in MALT and it is conceivable that B cell-specific deletion of TLR9 may affect mucosal immunity and change the microbiota structure. Thus, we hypothesize that alteration of TLR9 in B cells may affect the composition of commensals and gut barrier function, which could modulate autoimmune diabetes development. To test our hypothesis, we took two approaches: antibiotic treatment of *Tlr9* ^fl/fl^ *Cd19*-Cre^+^ NOD mice and adoptive transfer of the gut microbiota of *Tlr9* ^fl/fl^ *Cd19*-Cre^+^ NOD mice to germ-free wildtype NOD mice. Our results revealed that the microbiota of *Tlr9* ^fl/fl^ *Cd19*-Cre^+^ NOD mice increased intestinal permeability while promoting IL-10^+^ Breg cells. We further confirmed that the IL-10^+^ Breg cells induced by the altered gut microbiota can directly delay and protect the mice from T1D development. Our study provides a novel link between TLR9 in B cells and immuno-regulatory function of gut microbiota, which supports the concept of a microbiota-targeted therapy for T1D. Our findings also suggest an important notion that the integrity of the intestinal barrier is likely a double-edged sword and the role of the intestinal barrier in T1D development is more complex than previously reported. Thus, it requires further and deeper understanding.

## Methods and materials

### Mice

Mice (female, mixed mice from different breeders chosen randomly) used in this study were kept in specific pathogen-free (SPF) facilities with a 12-hour-light/dark (light: 7 am - 7 pm) cycle at the Yale Animal Resource Center (YARC). B-cell–specific TLR9-deficient (*Tlr9^f^* ^l/fl^ *Cd19*-Cre^+^, KO group) and the control (*Tlr9* ^fl/fl^ *Cd19*-Cre^−^, Ctr group) NOD mice were generated as previously reported (8). NOD mice, *Rag1* ^-/-^ NOD mice and *BDC2.5^+^* NOD mice were originally obtained from the Jackson Laboratory and maintained at SPF animal room at YARC. All the mice received autoclaved food (Global 2018S, Envigo), bedding and hyperchlorinated (4–6 ppm) water ad libitum. Germ-free (GF) NOD mouse breeders were generously provided by Dr. Kathy McCoy (University of Calgary, Canada) and have been bred and maintained at the gnotobiotic facility of YARC on 12-h light/dark cycles. The use of mice in this study was approved by the Institutional Animal Care and Use Committee at Yale University.

### Adoptive immune cell transfer and diabetes development

Total splenocytes from *BDC2.5^+^* NOD mice were injected (i.v.) into *Rag1* ^-/-^ NOD mice (5 × 10^6^/mouse). The recipients were monitored for glycosuria every 3 days, for 75 days. To circumvent the circadian rhythmic effect on blood glucose in mice, tail vein blood was measured daily at 9 a.m. using a glucometer (Bayer Diastix). Hyperglycemia was confirmed by two blood glucose measurements, 24 h apart, of over 250 mg/dl (>13.9 mmol/L) (26, 27).

### Intestinal permeability assay *in vivo*

Intestinal permeability was assessed based on the permeability to 4kD-FITC-dextran in plasma (DX-4000-FITC) (46944; Sigma-Aldrich, St. Louis, Missouri, USA) as previously described (19). Briefly, after fasting for 12 h, the mice were administered with DX-4000-FITC (500 mg/kg body weight, 125 mg/mL) by oral gavage. After 4 h, blood samples were collected, from the retro-orbital vein at different time points, diluted with an equal volume of PBS (pH 7.4) and analyzed for DX-4000-FITC concentration with a fluorescence spectrophotometer (HTS-7000 Plus-plate-reader; Perkin Elmer, Wellesley, Massachusetts, USA) at an excitation wavelength of 485 nm and emission wavelength of 535 nm. A standard curve was obtained by diluting known concentrations of FITC-dextran with normal plasma diluted with PBS (1:3 *v/v*).

### Histopathology

*Rag1* ^-/-^ NOD mice were transplanted with the fecal microbiota of *Tlr9* ^fl/fl^ *Cd19*-Cre^+^ (or control) NOD mice prior to transferring diabetogenic splenocytes. Pancreata were harvested from diabetic recipients and formalin-fixed and embedded in paraffin. Tissue sections were stained with hematoxylin and eosin. Insulitis was scored under light microscopy. Approximately 130 islets from 7 mice were individually scored blindly. The degree of lymphocyte infiltration was evaluated using a light microscope (Olympus, Ballerup, Denmark) and graded as follows: 0, no infiltration; 1, intact islets but with a few mononuclear cells surrounding the islets; 2, peri-insulitis; 3, islet infiltration <50%; 4, islet infiltration >50%.

### Cytokine ELISA

Murine IL-10 from the serum samples and splenocyte culture supernatants were measured using the Mouse ELISA kit (BioLegend), following the manufacturer’s instructions. The serum samples were diluted 1x with PBS (with 5% BSA), whereas the culture supernatants were measured without dilution.

### Antibiotic treatment *in vivo*

For depletion of endogenous commensal microbiota, *Tlr9* ^fl/fl^ *Cd19*-Cre^+^ mice, the control (*Tlr9* ^fl/fl^ *Cd19*-Cre^−^) NOD mice and *Rag1* ^-/-^ NOD mice were treated with an antibiotic cocktail (containing 0.5 g/L vancomycin, 1 g/L ampicillin, 1 g/L metronidazole, and 1 g/L neomycin) added in drinking water for 3 weeks. To mask the bitter taste of the added antibiotic cocktail, we added 0.1% energy-free sweetener to the drinking water.

### Collection of gut flush

The solid luminal contents from the mouse small intestine (jejunum and ileum) and colon were gently squeezed out followed by carefully flushing the intestine with 10 mL of precooled PBS (pH:7.2). After vortex, the suspensions were centrifuged at 8000 g for 15 minutes at 4°C. To minimize protein degradation, the clear supernatant was immediately transferred to cryovials and stored at -80°C. Immunoglobulin assessement was performed promptly to ensure sample integrity.

### Antibody measurements

Gut flush and serum samples were collected from the mice studied at termination. Antibody concentrations were determined by ELISA, using reagents purchased from Southern Biotech (Birmingham, AL), following the protocol previously described (28). Samples were diluted (gut flush 1:2–1:10, serum 1:100) before the antibody (IgA, IgG1, IgG2a and IgM) measurements. Antibody concentrations were converted according to standard curves.

### Extraction of gut bacterial DNA

Fresh fecal samples collected from the mice were resuspended in 300 μl Tris-EDTA buffer (10 mM Tris and 1 mM EDTA, pH8) containing 7.5 μl 0.5% SDS and 3 μl Proteinase K (200 μg/ml). The samples were then incubated at 37°C for 1 h followed by homogenization in a solution containing one volume of phenol/chloroform/isoamyl alcohol (25:24:1), 200 μl 20% SDS and 0.3 g zirconium silica beads, with a mini-bead-beater (BioSpec) for 2 minutes. Phenol/chloroform/isoamyl alcohol was then added to the samples prior to centrifugation (4°C, 12000 g, 15 mins). The upper aqueous layer, containing DNA, was transferred to a new tube. Bacterial DNA was subsequently precipitated with isopropanol, washed with 70% ethanol, air-dried, and resuspended in 100 μl of nuclease-free water.

### Bacterial load in the fecal samples

A 466 bp length sequence located in 16S rRNA gene was used for quantitative PCR (qPCR) with the primers Uni331F (TCCTACGGGAGGCAGCAGT) and Uni797R (GGACTACCAGGGTATCTAATCCTG TT) (29). Briefly, qPCR was performed with an iCycler qPCR machine (BIO-RAD, Geneva, Switzerland) using iQ SYBR Green Supermix (170-8882AP, BIO-RAD, CA, USA). The qPCR conditions were 95°C for 3 min, followed by 40 cycles of 95°C for 15 s, 60°C for 60 s and the plates were read for 5 s at 80°C. The bacterial load in the fecal samples was determined by a two color Real-Time PCR detection system (MyiQ2, BIO-RAD, USA).

### 16S rRNA gene sequencing and data analysis

The V4 region of the bacterial 16S rRNA gene was amplified from each DNA sample by PCR using barcoded broadly-conserved primer pairs (5’-GTGCCAGCMGCCGCGGTAA-3’) and (5’-GGACTACHVGGGTWTCTAAT-3’). The PCR products were purified using gel extraction kits (QIAGEN) and quantified by a Nanodrop spectrophotometer. Equimolar amounts of each sample were pooled for pyrosequencing using the Ion Torrent Personal Genome Machine (PGM) sequencing system (Life Technologies). The sequencing results were analyzed with the Quantitative Insights Into Microbial Ecology (QIIME) software package (version 1.8) and UPARSE pipeline (version 7.0). Taxonomy assignment was performed at various levels using representative sequences of each operational taxonomic unit (OTU). All the samples were rarefied to 10,000 reads per sample for downstream analysis. Principal coordinate analysis (PCoA) of OTUs based on Bray-Curtis distance was performed using QIIME. The statistical significance was subsequently assessed using permutational multivariate analysis of variance (PERMANOVA) with 9,999 permutations and P values were adjusted for multiple comparison with Benjamini-Hochberg method (30).

Sparse partial least-squares discriminant analysis (sPLS-DA) models (31) were established to identify specific OTUs that contributed to the segregation of gut microbial structure in control and KO mice using “mixOmics (v6.3.1)” package (32) in R (v3.4.4). Centered log ratio (CLR) transformations of the relative abundance of OTUs were implemented in sPLS-DA models. The optimal classification performances of the sPLS-DA models were estimated by the perf function using 5-fold cross-validation with the smallest balanced error rate.

### Bacterial stimulation of immune cells *in vitro*

Fresh fecal samples, collected from 6~8-week-old *Tlr9* ^fl/fl^ *Cd19*-Cre^+^ NOD mice and the *Tlr9* ^fl/fl^ *Cd19*-Cre^−^ NOD mice, were resuspended in sterile PBS (1 g/ml), and homogenized by vortexing vigorously for 30 secs. The samples were filtered through a 100 μm filter to remove large debris. Bacteria were re-suspended in sterile PBS and co-cultured overnight (10^5^∼10^9^ CFU) with 2.5 million total splenocytes from NOD mice. Stimulated splenocytes were further analyzed by flow cytometry after staining with different monoclonal antibodies conjugated with various flurochomes; all from BioLegend including CD1d (Clone: 1B1), B220 (Clone: RA3-6B2), CD5 (Clone: 53-7.3).

### Real time quantitative PCR

RNA from ileum and colon was extracted using Trizol reagent and a RNeasy mini plus kit (QIAGEN). After quantification, RNA was used for cDNA synthesis using the iScript cDNA synthesis kit (Invitrogen). Samples were analyzed on an iCycler qPCR machine (Bio-Rad). Gene expression level was determined using the 2^−ΔΔCt^ method and normalized with the reference gene, *Gapdh*. Primer sequences are listed in **Table 1**.

**Table 1:** The Primer Sequences of qPCR.

Genes	Primers	Sequence
*Muc1*	Forward	F-TACCCTACCTACCACACTCACG
	Reverse	R-CTGCTACTGCCATTACCTGC
*Muc2*	Forward	F-CACCAACACGTCAAAAATCG
	Reverse	R-GGTCTCTCGATCACCACCAT
*Muc3*	Forward	F-CTTCCAGCCTTCCCTAAACC
	Reverse	R-TCCACAGATCCATGCAAAAC
*Muc4*	Forward	F-GAGAGTTCCCTGGCTGTGTC
	Reverse	R-GGACATGGGTGTCTGTGTTG
*Zo-1*	Forward	F-ACCCGAAACTGATGCTGTGGATAG
	Reverse	F-ACCCGAAACTGATGCTGTGGATAG
*Occludin*	Forward	F- ATGTCCGGCCGATGCTCTC
	Reverse	R-TTTGGCTGCTCTTGGGTCTGTAT
*Cldn1*	Forward	F- GATGTGGATGGCTGTCATTG
	Reverse	R- CCTGGCCAAATTCATACCTG
*Cldn2*	Forward	F- TCATGCCCACCACAGAGATA
	Reverse	R- TATGTTGGTGCCAGCATTGT
*Cramp*	Forward	F- CTGCCCCCATACACTGCTTCAC
	Reverse	R- CCGAGCTGTGGATGACTTCA
*Reg3r*	Forward	F- TTCCTGTCCTCCATGATCAAAA
	Reverse	R- CATCCACCTCTGTTGGGTTCA
*b-defen*	Forward	F- AAGTACAGCACACCGGCCAC
	Reverse	R- GTATTCCTCATCTTGTTCTTGG
*Reg3b*	Forward	F- CTGCCTTAGACCGTGCTTTC
	Reverse	R- CCCTTGTCCATGATGCTCTT
*Relmb*	Forward	F- AGCTCTCAGTCGTCAAGAGCCTAA
	Reverse	R- CACAAGCACATCCAGTGACAACCA
*Defcr6*	Forward	F- CAGGCTGTGTCTGTCTCTTTTG
	Reverse	R- TAAATGACCCTTTCTGCAGGTC
*Gapdh*	Forward	F- GGCATGGACTGTGGTCATGAG
	Reverse	R- TGCACCACCAACTGCTTAGC

### Flow cytometry

Immune cells were incubated with an Fc-blocking antibody at 4°C for 15 min. Post- Fc-receptor blocking, cells were stained for surface markers using antibodies (conjugated with different fluorochromes, all from BioLegend) against CD1d (Clone: 1B1, Cat: 123524, BioLegend), CD40 (Clone: 3/23, Cat: 124626, BioLegend), B220 (Clone: RA3-6B2, Cat: 103224, BioLegend), CD5 (Clone: 53-7.3, Cat: 100624, BioLegend) and a viability dye (Zombie dye, BioLegend), for 30 min at 4°C. For intracellular cytokine staining, cells were incubated at 37°C in the presence of PMA (50 ng/mL, Sigma), Ionomycin (0.5 μg/mL, Sigma) and Golgi Plug (BD) for 4 h prior to washing and surface staining as outlined above. After surface staining, the cells were fixed (20 min, room temperature) and permeabilized (buffers purchased from BD Bioscience) and incubated with an Fc-blocking antibody at 4°C for 15 min prior to staining with anti-cytokine antibodies (30 min, 4°C) and washing. The cells were analyzed on a BD LSR II flow cytometry followed by analysis using Flowjo software.

### Statistics

Statistical analysis was performed using GraphPad Prism 9 software. Diabetes incidence was compared using log-rank test. *In vitro* assays were analyzed with Student’s t test or ANOVA, and P < 0.05 was considered to be statistically significant. The significance of beta diversity in gut microbiota was assessed using PerMANOVA. Differences in insulitis between two groups were evaluated using the chi-square test. The log-rank (Mantel-Cox) test was employed to examine differences in the incidence of type 1 diabetes (T1D) between two groups of mice.

## Results

### Microbiota-dependent gut barrier disruption in B-cell-specific TLR9-deficient NOD mice

**Figure 1:**
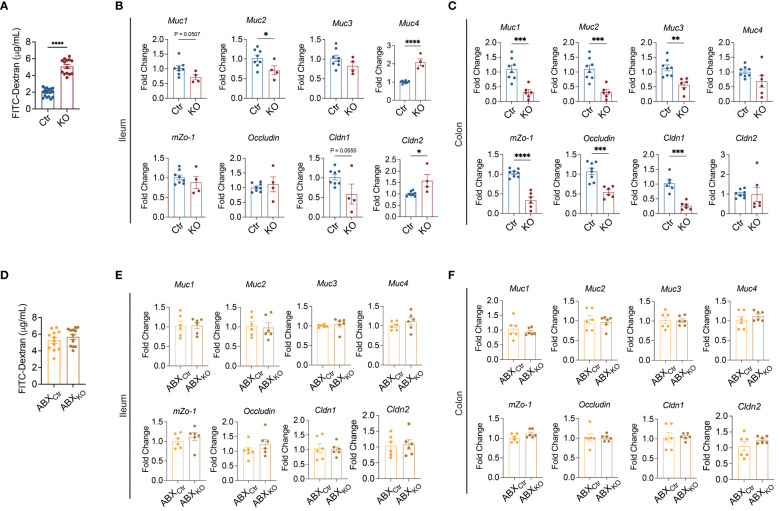
Microbiota-dependent gut barrier disruption in B cell-specific TLR9-deficient NOD mice. **(A)** FITC-dextran assessment of the permeability of gut barrier, Ctr group, n=17; KO group, n=14. **(B)** Levels of mRNA expression of the genes related to gut barrier integrity in the ileum tissue, Ctr group, n=8; KO group, n=4-6. **(C)** Levels of mRNA expression of genes related to gut barrier integrity in the colon tissue, Ctr group, n=8; KO group, n=6. **(D)** FITC-dextran assessment of the gut barrier permeability after antibiotic treatment. ABX_Ctr_ group, n=12; ABX_KO_ group, n=12. **(E)** Levels of mRNA expression of genes related to gut barrier integrity in the ileum tissue, ABX_Ctr_ group, n=6; ABX_KO_ group, n=6. **(F)** Levels of mRNA expression of genes related to gut barrier integrity in the colon tissue, Ctr and KO groups, ABX_Ctr_, n=6; ABX_KO_, n=6. The data in **(A–F)** are shown as the mean ± SEM, and Student’s t-test (two-tailed) was used to analyze the following pairs of groups: Ctr vs. KO, or ABX_Ctr_ vs. ABX_KO_. *P <0.05, **P <0.01, ***P <0.001 and ****P <0.0001. Ctr, *Tlr9* ^fl/fl^ *Cd19*-Cre^-^ NOD mice as control mice; KO, *Tlr9* ^fl/fl^ *Cd19*-Cre^+^ NOD mice; ABX_Ctr_, antibiotic cocktail-treated *Tlr9* ^fl/fl^ *Cd19*-Cre^-^ NOD mice; ABX_KO_, antibiotic cocktail-treated *Tlr9* ^fl/fl^ *Cd19*-Cre^+^ NOD mice.

We reported previously that B-cell-specific TLR9-deficient NOD mice were protected from autoimmune diabetes development (8). However, the role that the gut microbiota play in the protection is not known, given the fact that B cells are a major type of immune cells in the MALT. We first assessed gut permeability of B-cell-specific TLR9-deficient NOD mice (*Tlr9* ^fl/fl^ *Cd19*-Cre^+^,designated KO group) and control *Tlr9* ^fl/fl^ *Cd19*-Cre^-^ NOD mice (designated control (Ctr) group) with fluorescein isothiocyanate (FITC)-dextran test. Our results showed that *Tlr9* ^fl/fl^ *Cd19*-Cre^+^ NOD mice had a significant increase in gut permeability compared to the control mice (**Figure 1A**). Next, we evaluated the expression of 8 different genes related to gut barrier integrity in different segments of intestine and found that approximately one-half (9/16) of the mRNA expression levels were significantly downregulated in the ileum and colon of mice in the KO group (**Figures 1B, C**). To investigate whether the gut microbiota contribute to the gut barrier disruption in the *Tlr9* ^fl/fl^ *Cd19*-Cre^+^ NOD mice, we treated the two groups of mice with a cocktail of antibiotics for 3 weeks to deplete the gut microbiota followed by assessement of gut permeability *in vivo* and intestinal barrier associated gene expression *in vitro*. With depletion of gut microbiota, *Tlr9* ^fl/fl^ *Cd19*-Cre^+^ (ABX_KO_) mice showed an indistinguishable level of gut permeability compared to that of *Tlr9* ^fl/fl^ *Cd19*-Cre^-^ (ABX_Ctr_) NOD mice (**Figures 1D-F**). The change of gut microbiome is likely related to the specific knockout of TLR9 in B cells, which affects the level of immunoglobulins represented by IgA in the intestine where B cells are abundantly present (**Supplementary Figure 1A**). In addition, different IgG isotypes were all reduced in the serum (**Supplementary Figures 1B–D**). Similar to the immunoglobulin levels in the intestine, circulating IgA and some isotypes of IgG were also reduced in *Tlr9* ^fl/fl^ *Cd19*-Cre^+^ mice (**Supplementary Figures 2A–E**). Unlike the intestinal IgM, circulating IgM was markedly reduced in in *Tlr9* ^fl/fl^ *Cd19*-Cre^+^ mice (**Supplementary Figure 2E**). Thus, the depletion of the gut microbiota altered the gut barrier in the *Tlr9* ^fl/fl^ *Cd19*-Cre^+^ NOD mice, indicating that the gut microbiota are important in maintaining homeostasis of intestinal permeability.

### Alteration of gut microbiota and reduction of antimicrobial peptide gene expression in B cell-specific TLR9-deficient NOD mice

**Figure 2:**
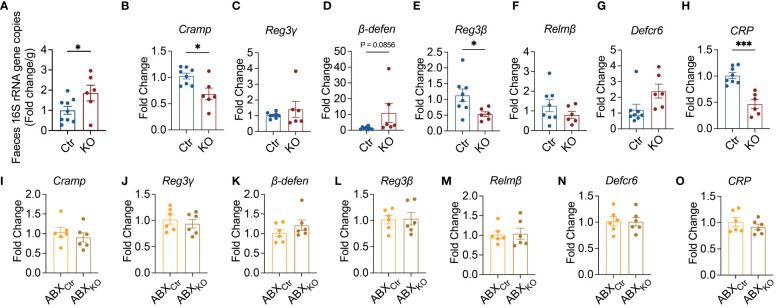
Microbiota-dependent suppression of antimicrobial peptide expression in B cell-specific TLR9-deficient NOD mice. **(A)** Quantification of bacterial load by qPCR of 16S rRNA gene in fecal samples, Ctr group, n=9; KO group, n=6. **(B-H)** Levels of mRNA expression of antimicrobial peptides in the colon tissue, Ctr group, n=8; KO group, n=6. **(I-O)** Effect of antibiotics on the levels of mRNA expression of antimicrobial peptides in the colon tissue, ABX_Ctr_ group, n=6; ABX_KO_ group, n=6. **(A-O)** are shown as the mean ± SEM, and Student’s t-test (two-tailed) was used to analyze the following pairs of groups: Ctr vs. KO, or ABX_Ctr_ vs. ABX_KO_. *P <0.05 and ***P <0.001. Ctr, *Tlr9* ^fl/fl^ *Cd19*-Cre^-^ NOD mice as control mice; KO, *Tlr9* ^fl/fl^ *Cd19*-Cre^+^ NOD mice; ABX_Ctr_, antibiotic cocktail-treated *Tlr9* ^fl/fl^ *Cd19*-Cre^-^ NOD mice; ABX_KO_, antibiotic cocktail-treated *Tlr9* ^fl/fl^ *Cd19*-Cre^+^ NOD mice.

To explore whether the altered mucosal immune milieu in Tlr9^fl/fl^ *Cd19*-Cre^+^ NOD mice affects the gut bacterial load, we quantified bacteria in fecal samples. Compared to the control mice, there was a significant increase in the bacterial load in the fecal samples from KO mice (**Figure 2A**). The maintenance of homeostasis of bacterial load in the intestine is influenced by different molecules, including immunoglobulins, mucins and antimicrobial peptides, all of which are related to intestinal mucosal immunity (33–35). Next, we quantified the gene expression of 7 different antimicrobial peptides in the colon, and found that the expression of *Cramp*, *Reg3b* and *CRP* was significantly down-regulated in the KO mice compared with the control mice (**Figures 2B–H**). We also observed the downregulation of *Cramp* in the ileum of the KO mice (**Supplementary Figure 3A**). Interestingly, under the gut microbiota-depleted conditions, treated by the antibiotic combination, there was no difference in the gene expression of all the antimicrobial peptides tested between the KO mice and the control mice in both colon and ileum (**Figures 2I–O**, **Supplementary Figure 4**). Thus, gut microbiota are required for regulating antimicrobial peptides in the intestinal tract of B cell-specific TLR9-deficient NOD mice.

### Enriched mucin-degrading Lachnospiraceae in *Tlr9* ^fl/fl^ *Cd19*-Cre^+^ NOD mice

**Figure 3:**
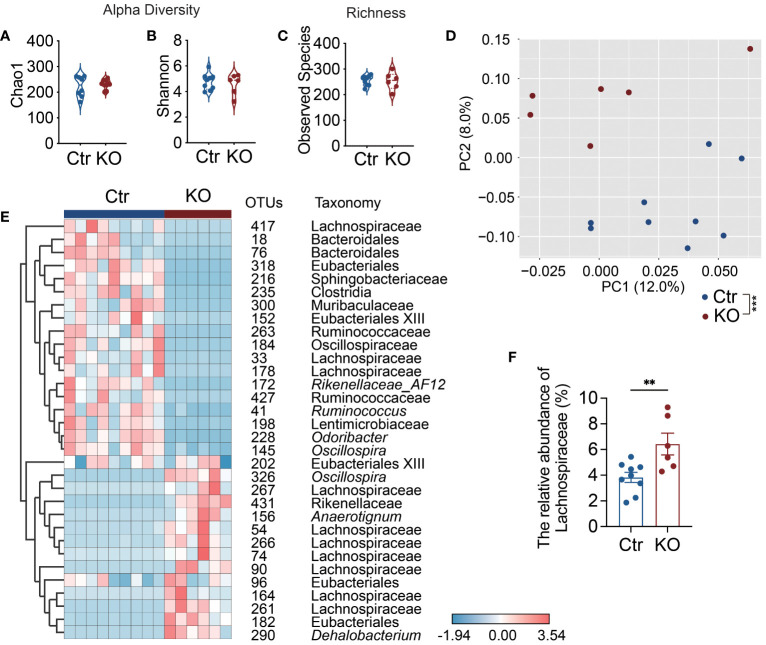
Lachnospiraceae are significantly enriched in *Tlr9* ^fl/fl^ *Cd19*-Cre^+^ NOD mice. **(A, B)** The alpha-diversity of gut microbiota in Ctr group and KO group mice, Ctr group, n=9; KO group, n=6. **(C)** The richness of gut microbiota in Ctr group and KO group mice, Ctr group, n=9; KO group, n=6. **(D)** Overall gut microbial structure in Ctr and KO group mice. Principal coordinate analysis (PCoA) was performed on the basis of Bray-Curtis distance at the operational taxonomic units (OTUs) level, Ctr group, n=9; KO group, n=6. **(E)** Thirty-two OTUs that were significantly different between Ctr and KO group, as identified using sPLS-DA models. The cluster tree on the left of the heat map shows associations between these OTUs, as determined by the Spearman correlation coefficient based on their relative abundances among all the samples. The heat map shows the relative abundance (log_10_ transformed) of each OTU in a sample from an individual mouse, Ctr group, n=9; KO group, n=6. **(F)** The relative abundance of Lachnospiraceae between Ctr and KO groups, Ctr group, n=9; KO group, n=6. The data in **(A–C, F)** are shown as the mean ± SEM, and Student’s t-test (two-tailed) was used to analyze difference between Ctr and KO groups. Permutational multivariate analysis of variance (PERMANOVA) test with 999 permutations was used to analyze difference between Ctr and KO groups in **(D)**. **P <0.01 and ***P <0.001. Ctr, *Tlr9* ^fl/fl^ *Cd19*-Cre^-^ NOD mice; KO, *Tlr9* ^fl/fl^ *Cd19*-Cre^+^ NOD mice.

To futher dissect the structure of the gut microbiota, we performed high-throughput sequencing of the 16S rRNA genes in the fecal samples of the mice from the two study groups. Although we did not find significant differences in alpha diversity and richness between the two groups of mice (**Figures 3A–C**), the structure of gut microbiota in the KO mice was significantly different from the control mice as seen in the principal coordinate analysis (PCoA) plot of Bray-Curtis distance (P=0.0001 with permutational multivariate analysis of variance (PERMANOVA) test (**Figure 3D**). Thus, in addition to altering bacterial load (**Figure 2A**), TLR9 deficiency in B cells resulted in significant changes in the gut microbiota in NOD mice.

To identify key members of the gut microbiota enriched in the KO mice that may contribute to the increased intestinal permeability and decreased antimicrobial peptides, we assessed bacteria load and found that there were significant differences between the *Tlr9* ^fl/fl^ *Cd19*-Cre^+^ and control mice in the operational taxonomic units (OTUs) of the gut microbiota using sparse partial least squares discriminant analysis (sPLS-DA) (**Figure 3E**; **Supplementary Figure 5**). Notable changes were observed in the family Lachnospiraceae (phylum Firmicutes), a major but understudied mammalian taxon (36). Specifically, 7 OTUs in this family were enriched, while 3 OTUs were reduced (**Figure 3E**). Taken together, our results revealed that Lachnospiraceae were significantly enriched in the *Tlr9* ^fl/fl^ *Cd19*-Cre^+^ NOD mice compared to the control mice (**Figure 3F**). Lachnospiraceae are known to have mucin-degrading properties (37); thus, the enriched Lachnospiraceae are likely to be related to the increased intestinal permeability in the KO mice.

### The adoptive transfer of gut microbiota from *Tlr9* ^fl/fl^ *Cd19*-Cre^+^ NOD mice into wild-type germ-free NOD mice recapitulates the *in vivo* phenotype of the *Tlr9* ^fl/fl^ *Cd19*-Cre^+^ NOD mice

**Figure 4:**
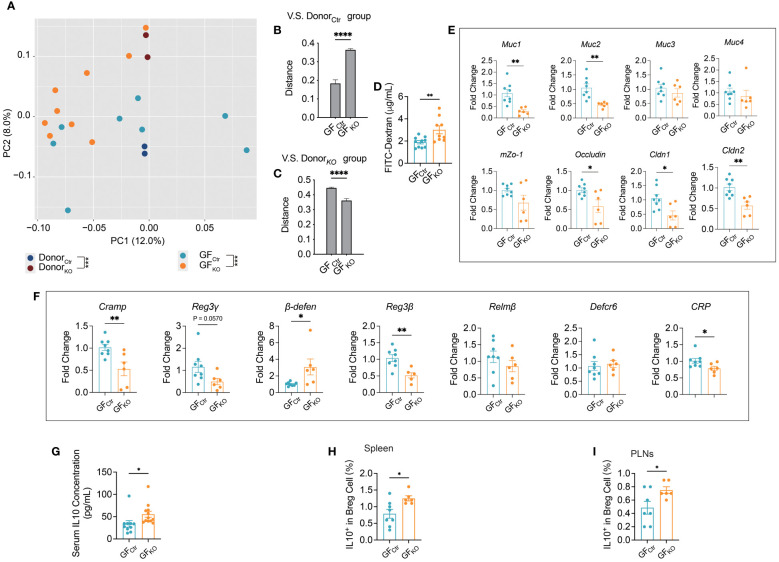
Germ-free (GF) wild type NOD mice mirrored the phenotype of *Tlr9* ^fl/fl^ *Cd19*-Cre^+^ NOD mice after transplanting the gut microbiome. **(A)** The overall structure of the gut microbiota of the Ctr, KO mouse donors, and GF mice transplanted with gut microbiota from Ctr and KO donors, and designated as GF_Ctr_ and GF_KO,_ respectively. Principal coordinate analysis (PCoA) was performed on the basis of Bray-Curtis distance at the operational taxonomic units (OTUs) level, Ctr donors (dark blue), n=2 batches of fecal samples pooled from n=20 mice; KO donors (dark purple), n=2 batches of fecal samples pooled from n=20 mice; GF_Ctr_ group (light blue), n=8; GF_KO_ (orange), n=9. **(B)** The distance between each recipient’s sample and each Ctr donor’s sample. **(C)** The distance between each recipient’s sample and each KO donor’s sample. **(D)** FITC-dextran assessment of the permeability of gut barrier one week after fecal microbiota transplantation, GF_Ctr_ group, n=10; GF_KO_ group, n=9. **(E)** Levels of mRNA expression of gut barrier integrity-related genes in the colon tissue one week after fecal microbiota transplantation, GF_Ctr_ group, n=8; GF_KO_ group, n=6. **(F)** Levels of mRNA expression of antimicrobial peptide genes in the colon tissue one week after fecal microbiota transplantation, GF_Ctr_ group, n=8; GF_KO_ group, n=6. **(G)** Serum IL-10 concentration, GF_Ctr_ group, n=10; GF_KO_ group, n=12. **(H, I)** IL10^+^ Breg cells in the spleens and pancreatic lymph nodes (PLNs) of GF recipient mice. GF_Ctr_ group, n=7-8; GF_KO_ group, n=6. The data in **(B-I)** are shown as the mean ± SEM, and Student’s t-test (two-tailed) was used to analyze the following pairs of groups: GF_Ctr_ vs. GF_KO_. *P <0.05, **P <0.01, ***P <0.001 and ****P <0.0001.

To test if the altered structure of gut microbiota in the KO mice was responsible for the gut mucosal changes seen in the KO mice, we transferred fresh feces from *Tlr9* ^fl/fl^ *Cd19*-Cre^+^ and the control *Tlr9* ^fl/fl^ *Cd19*-Cre^-^ NOD mice to wild-type germ-free (GF) NOD mice. The recipient mice were designated GF_KO_ and GF_Ctr_ mice, respectively. We found that the structures of gut microbiota in the GF_KO_ and GF_Ctr_ mice were close to their donors as early as one week after the fecal transplantation (**Figure 4A**; **Supplementary Figures 6A–C**). Quantification of the composition distance between the samples further confirmed this finding (**Figures 4B, C**). Next, we tested gut permeability of the receipient GF_KO_ and GF_Ctr_ mice with the FITC-dextran assay and observed a significant increase in gut permeability in GF_KO_ mice compared to the GF_Ctr_ mice (**Figure 4D**). The increased gut permeability was maintained in GF_KO_ mice one month after the fecal transplantation (**Supplementary Figure 7**). Our results indicated that the gut microbiota of *Tlr9* ^fl/fl^ *Cd19*-Cre^+^ NOD mice had a legacy effect on increasing intestinal permeability. Similar to the phenotype of the donor KO mice, there was a significant downregulation in the mRNA expression levels of five out of eight genes associated with gut barrier integrity in the colon of the GF_KO_ mice (**Figure 4E**). *Muc1* and *Cldn1* genes were also significantly downregulated in the ileum of GF_KO_ mice compared to the GF_Ctr_ mice (**Supplementary Figures 8A, G**). Among the detected genes related to the expression of antimicrobial peptides, *Cramp*, *β-defen* and *Reg3β* were significantly down-regulated in the the colon of GF_KO_ mice compared to those of GF_Ctr_ mice (**Figure 4F**), and the expression of *Reg3γ* appeared to be down-regulated (**Figure 4F**). Thus, our results showed the legacy effects of the gut microbiota on the regulation of gut barrier and antimicrobial peptides in the colon and ileum of the secondary hosts (**Supplementary Figures 8I–X**) and gut barrier related gene expression was further reduced with time in ileum (**Supplementary Figures 8Q–X**).

We have previously reported that TLR9 deficiency in B cells reduces pro-inflammatory signaling pathways by upregulating interleukin-10 (IL-10) secretion and this reduces the incidence of T1D in NOD mice (8). Considering that the microbiota modulate immune responses, directly or indirectly, we hypothesize that the gut microbiota of *Tlr9* ^fl/fl^ *CD19*-Cre^+^ NOD mice may directly promote IL-10 secretion. To test this hypothesis, we assessed circulating IL-10 of the recipient GF mice. Consistent with the donor *Tlr9* ^fl/fl^ *CD19*-Cre^+^ mice in our previous report (8), GF NOD mice that received gut microbiota from *Tlr9* ^fl/fl^ *Cd19*-Cre+ mice (GF_KO_) also had a higher levels of IL-10 in serum compared with GF_Ctr_ group (**Figure 4G**). Moreover, GF_KO_ mice showed a significantly increased proportion of IL-10-expressing Breg cells (regulatory B cells) in both the spleen and pancreatic lymph nodes (PLNs) (**Figures 4H, I**). Thus, the gut microbiota from *Tlr9* ^fl/fl^ *Cd19*-Cre^+^ NOD mice induced increased intestinal permeability, decreased expression of intestinal antimicrobial peptides and increased IL-10-expressing Bregs together with circulating IL-10.

### The gut microbiota of *Tlr9* ^fl/fl^ *Cd19*-Cre^+^ NOD mice directly promote Breg cell differentiation and IL-10 secretion *in vitro*

**Figure 5:**
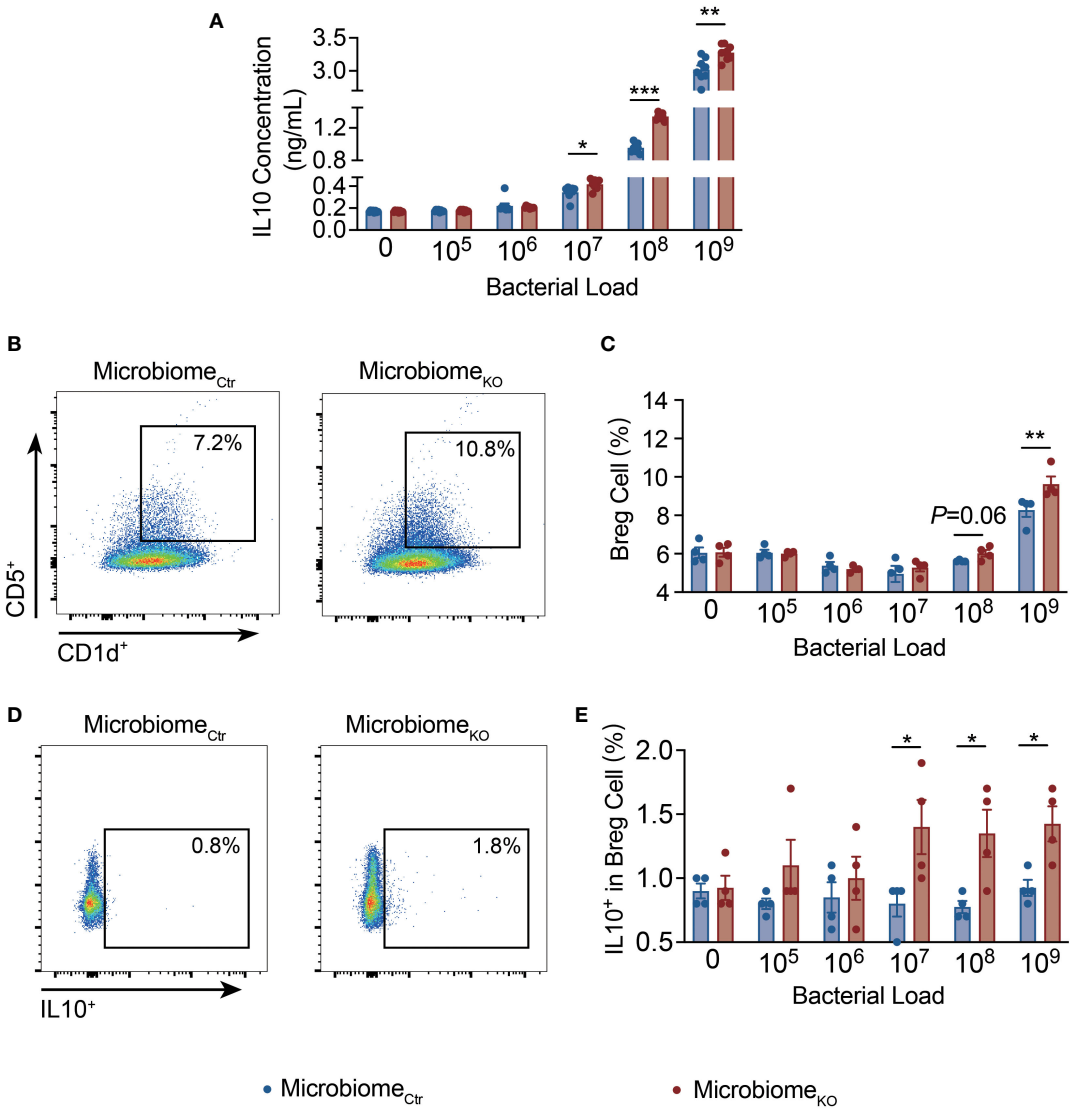
The Gut microbiota of *Tlr9* ^fl/fl^ *Cd19*-Cre^+^ NOD mice promote IL-10-producing Breg cells directly. **(A)** IL-10 concentrations in the supernatant of splenocytes of the NOD mice co-cultured with fecal bacteria from Ctr and KO groups, Microbiome_Ctr_ group, n=4; Micrombiome_KO_ group, n=4. Proportions of **(B, C)** CD5+CD1d+ splenic Breg cells, **(D, E)** IL-10^+^ splenic Breg cells of the NOD mice co-cultured with fecal bacteria from Ctr and KO groups, Microbiome_Ctr_ group, n=4; Micrombiome_KO_ group, n=4. The data in **(A, C, E)** are shown as the mean ± SEM, and Student’s t-test (two-tailed) was used to analyze the following pairs of groups: Microbiome_Ctr_ vs. Microbiome_KO_. *P <0.05, **P <0.01 and ***P <0.001. Microbiome_Ctr_. Splenocytes of NOD mice were co-cultured with fecal bacteria, at different concentrations, from Ctr and KO group mice, respectively, for 10 hours prior to the assays.

To further explore the direct effect of the microbiota of *Tlr9* ^fl/fl^ *Cd19*-Cre^+^ NOD mice on IL-10 related immune cells, we co-cultured splenocytes of wild type NOD mice with different concentrations of filtered gut bacteria for 10 hours (38). The culture supernatants were tested for secreted IL-10 by ELISA. Supporting the *in vivo* data from ex-GF NOD mice, we found that splenocytes stimulated with fecal bacteria from *Tlr9* ^fl/fl^ *Cd19*-Cre^+^ NOD mice promoted higher levels of IL-10 secretion in a dose dependent manner (**Figure 5A**). Notably, the proportion of Breg cells, assessed by flow cytometry, was in line with the profile of secreted IL-10 but at a higher bacterial dose (**Figures 5B, D**). Further, there were increased IL-10-secreting Breg cells (**Figures 5C, E**) whereas there was no difference in IL-10-produing T cells (data not shown). Thus, our results provided evidence that the fecal microbiota from *Tlr9* ^fl/fl^ *Cd19*-Cre^+^ NOD mice promoted IL-10-producing Breg cells directly.

### The gut microbiota of *Tlr9* ^fl/fl^ *Cd19*-Cre^+^ NOD mice protected these mice from development of autoimmune diabetes

**Figure 6:**
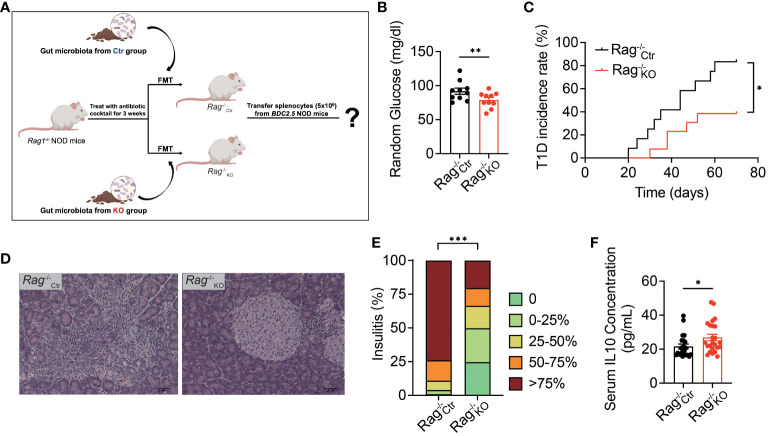
Gut microbiota of *Tlr9* ^fl/fl^ *Cd19*-Cre^+^ NOD mice protect antibiotic treated immuno-deficient *Rag* ^-/-^ NOD mice from T1D development. **(A)** Experimental flow diagram and the cocktail antibiotic-treated *Rag* ^-/-^ mice as pseudo germ-free *Rag1* ^-/-^ NOD mice. *Rag* ^-/-^_Ctr_, pseudo germ-free *Rag1* ^-/-^ NOD mice transplanted with fecal microbiota from the Ctr group; *Rag* ^-/-^_KO_, pseudo germ-free *Rag1* ^-/-^ NOD mice transplanted with fecal microbiota from the KO group. **(B)** Random blood glucose at 9 am from *Rag* ^-/-^_Ctr_ and *Rag* ^-/-^_KO_ mice. **(C)** T1D incidence (The diagnostic criterion for diabetes is a blood glucose level exceeding 250 mg/dl), *Rag* ^-/-^_Ctr_ group, n=11; *Rag* ^-/-^_KO_ group, n=12. **(D)** H&E-stained histological sections of pancreatic tissue (400x, scale bar = 0.05 mm) from *Rag* ^-/-^_Ctr_ mice and *Rag* ^-/-^_KO_ mice. **(E)** Insulitis score of *Rag* ^-/-^_Ctr_ and *Rag* ^-/-^_KO_ recipients. Islets were graded using the following scale: 1: No insulitis, 2: >25% insulitis, 3: 25–50% insulitis, 4: >50% insulitis, *Rag* ^-/-^_Ctr_ group, n=7; *Rag* ^-/-^_KO_ group, n=7. **(F)** Serum IL-10 concentration of the *Rag1* ^-/-^ NOD recipients: *Rag* ^-/-^_Ctr_ group, n=11; *Rag* ^-/-^_KO_ group, n=12. Data in **(C)** were assessed for significance using log-rank (Mantel-Cox) test. The data in **(B, F)** are shown as the mean ± SEM, and Student’s t-test (two-tailed) was used to analyze the following pairs of groups: *Rag* ^-/-^_Ctr_ vs. *Rag* ^-/-^_KO_. *P <0.05, **P <0.01. Data in **(E)** were analyzed with χ^2^ and ***P <0.001.

Our previous work showed that TLR9 deficiency in B cells delayed the onset of autoimmune diabetes in NOD mice (8). However, the role of the gut microbiota in diabetes protection was not known. To test whether the microbiota of *Tlr9* ^fl/fl^ *Cd19*-Cre^+^ NOD mice play a role in the disease protection, we performed an immune cell adoptive transfer approach using *Rag1* ^-/-^ NOD mice that were depleted of endogenous gut microbiota with antibiotic treatment(“pseudo germ-free” mice). The pseudo germ-free *Rag1* ^-/-^ NOD mice were colonized with gut microbiota from *Tlr9* ^fl/fl^ *Cd19*-Cre^+^ NOD mice (KO group) or and *Tlr9* ^fl/fl^ *Cd19*-Cre^-^ NOD mice (Ctr group), respectively, followed by adoptively transferring total splenocytes (5 × 10^6^/mouse) from *BDC2.5^+^* TCR transgenic NOD mice (**Figure 6A**) (39). The recipient mice were denoted *Rag^-/-^*_KO_ and *Rag1^-/-^*_Ctr_ mice, respectively. One week after the adoptive transfer, we assessed random blood glucose levels of the recipient mice. The results indicated that *Rag1* ^-/-^ KO mice colonized with microbiota from Tlr9^fl/fl^ Cd19-Cre^+^ donors (*Rag1^-/-^*_KO_) exhibited significantly lower blood glucose levels compared to the *Rag1* ^-/-^ mice that were colonized with gut microbiota from Tlr9^fl/fl^ Cd19-Cre^-^ control donors (*Rag1^-/-^*_Ctr_, **Figure 6B**). Moreover, the incidence of diabetes in the *Rag1^-/-^*_KO_ mice was significantly delayed and reduced compared with the *Rag1^-/-^*_Ctr_ mice (**Figure 6C**). Supporting the findings related to diabetes development, *Rag1^-/-^*_KO_ mice also had significantly less severe insulitis compared to the *Rag1^-/-^*_Ctr_ mice (**Figures 6D, E**). Next we assessed the anti-inflammatory cytokine IL-10 in the circulation and found much higher IL-10 levels in the serum samples from the *Rag1^-/-^*_KO_ mice compared with the *Rag^-/-^*_Cre_ mice (**Figure 6F**). Thus, our results indicated that the microbiota of *Tlr9* ^fl/fl^ *Cd19*-Cre^+^ NOD mice are directly involved in diabetes protection by modulating the host immune tolerance through promoting the secretion of immune-regulatory cytokine IL-10.

## Discussion

Our prior work showed IL-10-producing B regulatory cells (Bregs) protected B cell-specific TLR9-deficient NOD mice from diabetes (8). However, the role of mucosal immunophysiology and gut microbiota in generating these protective IL-10-producing Bregs was unclear. Given B cells, which have high TLR9 expression (40–42), are one of the major mucosal immune cells and gut microbiota’s potential pathogenic role in T1D (16, 43–45), we hypothesized that gut microbiota induce IL-10 elevation in B cell-specific TLR9-deficient NOD mice. To test this, we sequenced *Tlr9* ^fl/fl^ *Cd19*-Cre^+^ NOD mice’s gut microbiota, revealing dominant mucin-degrading Lachnospiraceae bacteria. Using germ-free NOD mice and antibiotic-treated *Tlr9* ^fl/fl^ *Cd19*-Cre^+^ NOD mice, we demonstrated that B cell-specific TLR9-deficiency altered microbiota, increased intestinal permeability and reduced antimicrobial peptide expression. These traits were reproduced in germ-free NOD mice and microbiota-depleted *Rag1* ^-/-^ NOD mice after transplanting gut microbiota from B cell-specific TLR9-deficient donors.

B-cell activation by TLR9 ligands is important for optimal antibody responses to microbial antigens and DNAs released from both physiological and pathological dying cells (46, 47). We showed here that B cell-specific TLR9-deficiency impaired class switched immunoglobulins, especially IgA, normally enriched in the intestinal lumen. Secretory IgA by B cells maintains intestinal microbiota homeostasis, which includes the relative taxonomy and bacterial load (48–50). TLR9-deficient B cells induced a decrease luminal immunoglobulins which could contribute to the increased bacterial load in the intestine, accompanied by the decreased intestinal antimicrobial peptides and increased intestinal permeability. However, depleting gut microbiota using a combination of antibiotics in *Tlr9* ^fl/fl^ *Cd19*-Cre^+^ NOD mice reversed the decreased intestinal antimicrobial peptides and increased intestinal permeability, suggesting that the these features were not dependent on luminal antibodies but rather on the gut microbiota. Furthermore, the transfer of feces of *Tlr9* ^fl/fl^ *Cd19*-Cre^+^ NOD mice to wild type germ-free NOD mice, indicated that the gut microbiota transferred the features of decreased gene expression of antimicrobial peptides and increased intestinal permeability seen in the donor *Tlr9* ^fl/fl^ *Cd19*-Cre^+^ NOD mice. In addition, we found that Lachnospiraceae, mucin degrading bacteria (51–54), were enriched in the gut of *Tlr9* ^fl/fl^ *Cd19*-Cre^+^ NOD mice. Thus, the increased mucin-degrading Lachnospiraceae and increased absolute bacterial load likely contribute to the increased intestinal permeability in *Tlr9* ^fl/fl^ *Cd19*-Cre^+^ NOD mice.

Previous studies in both animals and humans suggested that increased gut permeability was associated with the development of Type 1 Diabetes (T1D) (15, 55). However, the lack of differences in intestinal permeability between diabetic NOD mice and their age-and gender-matched non-diabetic counterparts indicates that changes in intestinal permeability are not an essential factor for the development of T1D (20). A low-dose chemical, Dextran sulfate sodium (DSS) that damages the intestinal barrier induced impaired glocose tolerance without affecting intestinal permeability (19). Lipopolysaccharide (LPS), derived mainly from Gram-negative bacteria damages the intestinal barrier (56), but LPS can also alleviate the development of T1D by promoting the differentiation of IL-10-positive immune cells (57–60). In this study, we found that the altered gut microbiota due to TLR9 deficiency in B cells increased intestinal permeability of the hosts, promoted the differentiation of IL-10^+^ Breg cells and protected the hosts from developing T1D. Although our finding does not establish a direct link between intestinal permeability and T1D onset, it underscores the complex nature of the disease and its multifactorial immunopathogenesis. Our previous study showed that *Tlr9* ^fl/fl^ *Cd19*-Cre^+^ NOD mice protected from T1D development by promoting IL-10-associated immune network (9), and in the current study, we revealed that the altered gut microbiota in *Tlr9* ^fl/fl^ *Cd19*-Cre^+^ NOD mice were the cause of promoting the IL-10^+^ Breg cells and protected the hosts from T1D development. Our study further supports link between the microbiome function and the host immune-metabolic phenotype. Thus, it is important to investigate the function of gut microbiome related to the etiology of T1D from the perspective of intestinal mucosal immunity and the autoimmune response. Further, intestinal integrity may be a concomitant phenomenon affected by other factors. The future direction of this study will be to isolate the bacterial strains that have immune regulatory function and can alleviate or assist in the treatment of T1D. Our current study provides a preclinical basis for the prevention from and/or treatment of T1D by targeting the gut microbiota.

## Data availability statement

The datasets presented in this study can be found in online repositories. The names of the repository/repositories and accession number(s) can be found below: https://ngdc.cncb.ac.cn/gsa/s/4teywtRM, CRA012538.

## Ethics statement

The animal study was approved by Institution Animal Care and Use Committee (IACUC), Yale University. The study was conducted in accordance with the local legislation and institutional requirements.

## Author contributions

XY: Conceptualization, Data curation, Funding acquisition, Investigation, Software, Validation, Writing – original draft, Writing – review & editing. JH: Investigation, Writing – review & editing. JP: Investigation, Writing – review & editing. PW: Investigation, Writing – review & editing. FW: Investigation, Writing – review & editing. RW: Funding acquisition, Software, Writing – review & editing. DW: Funding acquisition, Writing – review & editing. LW: Conceptualization, Funding acquisition, Supervision, Validation, Writing – review & editing.

## References

[1] DiMeglio LA Evans-Molina C Oram RA. Type 1 diabetes. Lancet. (2018) 391:2449–62. doi: 10.1016/S0140-6736(18)31320-5 PMC6661119 29916386

[2] Kim TK Lee MS. Innate immune receptors in type 1 diabetes: the relationship to cell death-associated inflammation. Biochem Soc Trans. (2020) 48:1213–25. doi: 10.1042/BST20200131 32510139

[3] Hemmi H Takeuchi O Kawai T Kaisho T Sato S Sanjo H. A Toll-like receptor recognizes bacterial DNA. Nature. (2000) 408:740–5. doi: 10.1038/35047123 11130078

[4] Liu Y Yin H Zhao M Lu Q. TLR2 and TLR4 in autoimmune diseases: a comprehensive review. Clin Rev Allergy Immunol. (2014) 47:136–47. doi: 10.1007/s12016-013-8402-y 24352680

[5] Guo Q Qu H Zhang H Zhong X. Prunella vulgaris L. Attenuates experimental autoimmune thyroiditis by inhibiting HMGB1/TLR9 signaling. Drug Des Devel Ther. (2021) 15:4559–74. doi: 10.2147/DDDT.S325814 PMC8576104 34764638

[6] Makita Y Suzuki H Kano T Takahata A Julian BA Novak J. TLR9 activation induces aberrant IgA glycosylation via APRIL- and IL-6-mediated pathways in IgA nephropathy. Kidney Int. (2020) 97:340–9. doi: 10.1016/j.kint.2019.08.022 PMC7372907 31748116

[7] Tai N Wong FS Wen L. TLR9 deficiency promotes CD73 expression in T cells and diabetes protection in nonobese diabetic mice. J Immunol. (2013) 191:2926–37. doi: 10.4049/jimmunol.1300547 PMC3788667 23956420

[8] Sha S Pearson JA Peng J Hu Y Huang J Xing Y. TLR9 deficiency in B cells promotes immune tolerance via interleukin-10 in a type 1 diabetes mouse model. Diabetes. (2021) 70:504–15. doi: 10.2337/db20-0373 PMC7881860 33154070

[9] Liu M Peng J Tai N Pearson JA Hu C Guo J. Toll-like receptor 9 negatively regulates pancreatic islet beta cell growth and function in a mouse model of type 1 diabetes. Diabetologia. (2018) 61:2333–43. doi: 10.1007/s00125-018-4705-0 PMC6182661 30094467

[10] Rewers M Ludvigsson J. Environmental risk factors for type 1 diabetes. Lancet. (2016) 387:2340–8. doi: 10.1016/S0140-6736(16)30507-4 PMC5571740 27302273

[11] Costa FR Francozo MC de Oliveira GG Ignacio A Castoldi A Zamboni DS. Gut microbiota translocation to the pancreatic lymph nodes triggers NOD2 activation and contributes to T1D onset. J Exp Med. (2016) 213:1223–39. doi: 10.1084/jem.20150744 PMC4925011 27325889

[12] Vatanen T Franzosa EA Schwager R Tripathi S Arthur TD Vehik K. The human gut microbiome in early-onset type 1 diabetes from the TEDDY study. Nature. (2018) 562:589–94. doi: 10.1038/s41586-018-0620-2 PMC6296767 30356183

[13] Vehik K Lynch KF Wong MC Tian XJ Ross MC Gibbs RA. Prospective virome analyses in young children at increased genetic risk for type 1 diabetes. Nat Med. (2019) 25:1865–+. doi: 10.1038/s41591-019-0667-0 PMC6898786 31792456

[14] Antvorskov JC Halldorsson TI Josefsen K Svensson J Granstrom C Roep BO. Association between maternal gluten intake and type 1 diabetes in offspring: national prospective cohort study in Denmark. Bmj-Brit Med J. (2018) 362:k3547. doi: 10.1136/bmj.k3547 PMC6283375 30232082

[15] Monsted MO Falck ND Pedersen K Buschard K Holm LJ Haupt-Jorgensen M. Intestinal permeability in type 1 diabetes: An updated comprehensive overview. J Autoimmun. (2021) 122:102674. doi: 10.1016/j.jaut.2021.102674 34182210

[16] Sorini C Cosorich I Lo Conte M De Giorgi L Facciotti F Luciano R. Loss of gut barrier integrity triggers activation of islet-reactive T cells and autoimmune diabetes. Proc Natl Acad Sci U S A. (2019) 116:15140–9. doi: 10.1073/pnas.1814558116 PMC6660755 31182588

[17] Li X Atkinson MA. The role for gut permeability in the pathogenesis of type 1 diabetes–a solid or leaky concept? Pediatr Diabetes. (2015) 16:485–92. doi: 10.1111/pedi.12305 PMC4638168 26269193

[18] Secondulfo M Iafusco D Carratu R deMagistris L Sapone A Generoso M. Ultrastructural mucosal alterations and increased intestinal permeability in non-celiac, type I diabetic patients. Dig Liver Dis. (2004) 36:35–45. doi: 10.1016/j.dld.2003.09.016 14971814

[19] Yang X Wang Z Niu J Zhai R Xue X Wu G. Pathobionts from chemically disrupted gut microbiota induce insulin-dependent diabetes in mice. Microbiome. (2023) 11:62. doi: 10.1186/s40168-023-01507-z 36978130 PMC10052834

[20] Hadjiyanni I Li KK Drucker DJ. Glucagon-like peptide-2 reduces intestinal permeability but does not modify the onset of type 1 diabetes in the nonobese diabetic mouse. Endocrinology. (2009) 150:592–9. doi: 10.1210/en.2008-1228 18845625

[21] Li N Hatch M Wasserfall CH Douglas-Escobar M Atkinson MA Schatz DA. Butyrate and type 1 diabetes mellitus: can we fix the intestinal leak? J Pediatr Gastroenterol Nutr. (2010) 51:414–7. doi: 10.1097/MPG.0b013e3181dd913a 20706153

[22] Groussin M Mazel F Alm EJ. Co-evolution and co-speciation of host-gut bacteria systems. Cell Host Microbe. (2020) 28:12–22. doi: 10.1016/j.chom.2020.06.013 32645351

[23] Blanton LV Charbonneau MR Salih T Barratt MJ Venkatesh S Ilkaveya O. Gut bacteria that prevent growth impairments transmitted by microbiota from malnourished children. Science. (2016) 351:6275. doi: 10.1126/science.aad3311 PMC4787260 26912898

[24] Ridaura VK Faith JJ Rey FE Cheng J Duncan AE Kau AL. Gut microbiota from twins discordant for obesity modulate metabolism in mice. Science. (2013) 341:1241214. doi: 10.1126/science.1241214 24009397 PMC3829625

[25] Smits LP Bouter KE de Vos WM Borody TJ Nieuwdorp M. Therapeutic potential of fecal microbiota transplantation. Gastroenterology. (2013) 145:946–53. doi: 10.1053/j.gastro.2013.08.058 24018052

[26] Pearson JA Tai N Ekanayake-Alper DK Peng J Hu Y Hager K. Norovirus changes susceptibility to type 1 diabetes by altering intestinal microbiota and immune cell functions. Front Immunol. (2019) 10:2654. doi: 10.3389/fimmu.2019.02654 31798584 PMC6863139

[27] Huang J Tan Q Tai N Pearson JA Li Y Chao C. IL-10 deficiency accelerates type 1 diabetes development via modulation of innate and adaptive immune cells and gut microbiota in BDC2.5 NOD Mice. Front Immunol. (2021) 12:702955. doi: 10.3389/fimmu.2021.702955 34394099 PMC8362616

[28] Li YY Pearson JA Chao C Peng J Zhang X Zhou Z. Nucleotide-binding oligomerization domain-containing protein 2 (Nod2) modulates T1DM susceptibility by gut microbiota. J Autoimmun. (2017) 82:85–95. doi: 10.1016/j.jaut.2017.05.007 28592385 PMC8284907

[29] Nadkarni MA Martin FE Jacques NA Hunter N. Determination of bacterial load by real-time PCR using a broad-range (universal) probe and primers set. Microbiol (Reading). (2002) 148:257–66. doi: 10.1099/00221287-148-1-257 11782518

[30] Anderson MJ. A new method for non-parametric multivariate analysis of variance. Austral Ecol. (2001) 26:32–46. doi: 10.1111/j.1442-9993.2001.01070.pp.x

[31] Le Cao KA Boitard S Besse P. Sparse PLS discriminant analysis: biologically relevant feature selection and graphical displays for multiclass problems. BMC Bioinf. (2011) 12:253. doi: 10.1186/1471-2105-12-253 PMC3133555 21693065

[32] Rohart F Gautier B Singh A Le Cao KA. mixOmics: An R package for 'omics feature selection and multiple data integration. PloS Comput Biol. (2017) 13:e1005752. doi: 10.1371/journal.pcbi.1005752 29099853 PMC5687754

[33] Kett K Baklien K Bakken A Kral JG Fausa O Brandtzaeg P. Intestinal B-cell isotype response in relation to local bacterial load: evidence for immunoglobulin A subclass adaptation. Gastroenterology. (1995) 109:819–25. doi: 10.1016/0016-5085(95)90389-5 7657110

[34] Macpherson AJ Geuking MB McCoy KD. Immune responses that adapt the intestinal mucosa to commensal intestinal bacteria. Immunology. (2005) 115:153–62. doi: 10.1111/j.1365-2567.2005.02159.x PMC1782138 15885120

[35] Cardoso MH Meneguetti BT Oliveira-Junior NG Macedo MLR Franco OL. Antimicrobial peptide production in response to gut microbiota imbalance. Peptides. (2022) 157:170865. doi: 10.1016/j.peptides.2022.170865 36038014

[36] Meehan CJ Beiko RG. A phylogenomic view of ecological specialization in the Lachnospiraceae, a family of digestive tract-associated bacteria. Genome Biol Evol. (2014) 6:703–13. doi: 10.1093/gbe/evu050 PMC3971600 24625961

[37] Hoskins LC Agustines M McKee WB Boulding ET Kriaris M Niedermeyer G. Mucin degradation in human colon ecosystems. Isolation and properties of fecal strains that degrade ABH blood group antigens and oligosaccharides from mucin glycoproteins. J Clin Invest. (1985) 75:944–53. doi: 10.1172/JCI111795 PMC423632 3920248

[38] Huang J Peng J Pearson JA Efthimiou G Hu Y Tai N. Toll-like receptor 7 deficiency suppresses type 1 diabetes development by modulating B-cell differentiation and function. Cell Mol Immunol. (2021) 18:328–38. doi: 10.1038/s41423-020-00590-8 PMC8027372 33432061

[39] Tan Q Tai N Li Y Pearson J Pennetti S Zhou Z. Activation-induced cytidine deaminase deficiency accelerates autoimmune diabetes in NOD mice. JCI Insight. (2018) 3:e95882. doi: 10.1172/jci.insight.95882 29321370 PMC5821212

[40] Saber MM Monir N Awad AS Elsherbiny ME Zaki HF. TLR9: A friend or a foe. Life Sci. (2022) 307:120874. doi: 10.1016/j.lfs.2022.120874 35963302

[41] Fillatreau S Manfroi B Dorner T. Toll-like receptor signalling in B cells during systemic lupus erythematosus. Nat Rev Rheumatol. (2021) 17:98–108. doi: 10.1038/s41584-020-00544-4 33339987 PMC7747191

[42] Suthers AN Sarantopoulos S. TLR7/TLR9- and B cell receptor-signaling crosstalk: promotion of potentially dangerous B cells. Front Immunol. (2017) 8:775. doi: 10.3389/fimmu.2017.00775 28751890 PMC5507964

[43] Wen L Ley RE Volchkov PY Stranges PB Avanesyan L Stonebraker AC. Innate immunity and intestinal microbiota in the development of Type 1 diabetes. Nature. (2008) 455:1109–13. doi: 10.1038/nature07336 PMC2574766 18806780

[44] Markle JG Frank DN Mortin-Toth S Robertson CE Feazel LM Rolle-Kampczyk U. Sex differences in the gut microbiome drive hormone-dependent regulation of autoimmunity. Science. (2013) 339:1084–8. doi: 10.1126/science.1233521 23328391

[45] Sun J Furio L Mecheri R van der Does AM Lundeberg E Saveanu L. Pancreatic beta-Cells Limit Autoimmune Diabetes via an Immunoregulatory Antimicrobial Peptide Expressed under the Influence of the Gut Microbiota. Immunity. (2015) 43:304–17. doi: 10.1016/j.immuni.2015.07.013 26253786

[46] Sindhava VJ Oropallo MA Moody K Naradikian M Higdon LE Zhou L. A TLR9-dependent checkpoint governs B cell responses to DNA-containing antigens. J Clin Invest. (2017) 127:1651–63. doi: 10.1172/JCI89931 PMC5409796 28346226

[47] Capolunghi F Rosado MM Cascioli S Girolami E Bordasco S Vivarelli M. Pharmacological inhibition of TLR9 activation blocks autoantibody production in human B cells from SLE patients. Rheumatology. (2010) 49:2281–9. doi: 10.1093/rheumatology/keq226 20739362

[48] Kogut MH Lee A Santin E. Microbiome and pathogen interaction with the immune system. Poult Sci. (2020) 99:1906–13. doi: 10.1016/j.psj.2019.12.011 PMC7587753 32241470

[49] Sender R Fuchs S Milo R. Are we really vastly outnumbered? Revisiting the ratio of bacterial to host cells in humans. Cell. (2016) 164:337–40. doi: 10.1016/j.cell.2016.01.013 26824647

[50] Sender R Fuchs S Milo R. Revised estimates for the number of human and bacteria cells in the body. PloS Biol. (2016) 14:e1002533. doi: 10.1371/journal.pbio.1002533 27541692 PMC4991899

[51] Png CW Linden SK Gilshenan KS Zoetendal EG McSweeney CS Sly LI. Mucolytic bacteria with increased prevalence in IBD mucosa augment *in vitro* utilization of mucin by other bacteria. Am J Gastroenterol. (2010) 105:2420–8. doi: 10.1038/ajg.2010.281 20648002

[52] Crost EH Tailford LE Le Gall G Fons M Henrissat B Juge N. Utilisation of mucin glycans by the human gut symbiont Ruminococcus gnavus is strain-dependent. PloS One. (2013) 8:e76341. doi: 10.1371/journal.pone.0076341 24204617 PMC3808388

[53] Jellbauer S Raffatellu M. An intestinal arsonist: pathobiont ignites IBD and flees the scene. Gut. (2014) 63:1034–5. doi: 10.1136/gutjnl-2013-305589 24026350

[54] Duck LW Walter MR Novak J Kelly D Tomasi M Cong Y. Isolation of flagellated bacteria implicated in Crohn's disease. Inflammation Bowel Dis. (2007) 13:1191–201. doi: 10.1002/ibd.20237 17712838

[55] Bielka W Przezak A Pawlik A. The role of the gut microbiota in the pathogenesis of diabetes. Int J Mol Sci. (2022) 23:480. doi: 10.3390/ijms23010480 35008906 PMC8745411

[56] Stephens M von der Weid PY. Lipopolysaccharides modulate intestinal epithelial permeability and inflammation in a species-specific manner. Gut Microbes. (2020) 11:421–32. doi: 10.1080/19490976.2019.1629235 PMC7524286 31203717

[57] Pearson JA Peng J Huang J Yu X Tai N Hu Y. NLRP6 deficiency expands a novel CD103(+) B cell population that confers immune tolerance in NOD mice. Front Immunol. (2023) 14:1147925. doi: 10.3389/fimmu.2023.1147925 36911699 PMC9995752

[58] Tian J Zekzer D Hanssen L Lu Y Olcott A Kaufman DL. Lipopolysaccharide-activated B cells down-regulate Th1 immunity and prevent autoimmune diabetes in nonobese diabetic mice. J Immunol. (2001) 167:1081–9. doi: 10.4049/jimmunol.167.2.1081 11441119

[59] Lampropoulou V Hoehlig K Roch T Neves P Calderon Gomez E Sweenie CH. TLR-activated B cells suppress T cell-mediated autoimmunity. J Immunol. (2008) 180:4763–73. doi: 10.4049/jimmunol.180.7.4763 18354200

[60] Boldison J Da Rosa LC Davies J Wen L Wong FS. Dendritic cells license regulatory B cells to produce IL-10 and mediate suppression of antigen-specific CD8 T cells. Cell Mol Immunol. (2020) 17:843–55. doi: 10.1038/s41423-019-0324-z PMC7395736 31728048

