# Supplementary material for: Gut microbiota from B-cell-specific TLR9-deficient NOD mice promote IL-10+ Breg cells and protect against T1D
Source: Front Immunol. 2024 Jun 6;15:1413177. doi: 10.3389/fimmu.2024.1413177 (PMC11187306; doi:10.3389/fimmu.2024.1413177)
Supplement: Supplementary file 1 [file DataSheet_1.docx]

**Supplementary Figures**

**Figure S1**

**Figure S1 | Immunoglobulin concentration in gut flush (the entire intestine). (A)** IgA, **(B)** IgG1, **(C)** IgG2a, and **(D)** IgM of Ctr and KO mice. Ctr group, n=5; KO group, n=6-7. The data in **(A)-(D)** are shown as the mean ± SEM, and Student’s t-test (two-tailed) was used to analyze the following pairs of groups: Ctr vs. KO. **P* <0.05, ***P* <0.01. Ctr, *Tlr9*^fl/fl^ *Cd19*-Cre^-^ NOD mice as control mice; KO, *Tlr9*^fl/fl^ *Cd19*-Cre^+^ NOD mice.

**Figure S2**

**Figure S2 | Immunoglobulin concentration in serum. (A)** IgA, **(B)** IgG1, **(C)** IgG2a, and **(D)** IgM of Ctr and KO mice. Ctr group, n=5; KO group, n=5. The data in **(A)-(E)** are shown as the mean ± SEM, and Student’s t-test (two-tailed) was used to analyze the following pairs of groups: Ctr vs. KO. **P* <0.05, ***P* <0.01. Ctr, *Tlr9*^fl/fl^ *Cd19*-Cre^-^ NOD mice as control mice; KO, *Tlr9*^fl/fl^ *Cd19*-Cre^+^ NOD mice.

**Figure S3**

**Figure S3 |** Levels of mRNA expression of antimicrobial peptide genes in the ileum of Ctr and KO mice, Ctr group, n=8; KO group, n=4. The data in **(A)-(G)** are shown as the mean ± SEM, and Student’s t-test (two-tailed) was used to analyze the following pairs of groups: Ctr vs. KO. ***P* <0.01. Ctr, *Tlr9*^fl/fl^ *Cd19*-Cre^-^ NOD mice as control mice; KO, *Tlr9*^fl/fl^ *Cd19*-Cre^+^ NOD mice.

**Figure S4**

**Figure S4 |** Levels of mRNA expression of antimicrobial peptide genes in the ileum of Ctr and KO mice after treatment with antibiotics (ABX). ABX_Ctr_, antibiotic cocktail-treated *Tlr9*^fl/fl^ *Cd19*-Cre^-^ NOD mice; ABX_KO_, antibiotic cocktail-treated *Tlr9*^fl/fl^ *Cd19*-Cre^+^ NOD mice. ABX_Ctr_ group, n=6; ABX_KO_ group, n=6. The data in **(A)-(G)** are shown as the mean ± SEM, and Student’s t-test (two-tailed) was used to analyze the following pairs of groups: ABX_Ctr_ vs. ABX_KO_.

**Figure S5**

**Figure S5 | Optimal classification performance of the sPLS-DA model of the gut microbial structure in Ctr and KO mice. (A)** sPLS-DA plot of the gut microbial structure. **(B)** Error rate of the sPLS-DA model. *Tlr9*^fl/fl^ *Cd19*-Cre^-^ NOD mice as control mice (Ctr); *Tlr9*^fl/fl^ *Cd19*-Cre^+^ NOD mice as knockout (KO). The optimal classification performances of the sPLS-DA models were estimated by the perf function using 5-fold cross-validation with the lowest balanced error rate.

**Figure S6**

**Figure S6 | The alpha-diversity of gut microbiota in germ-free (GF) NOD mice transplanted with fecal microbiota from control and knockout mice.** GF_Ctr_, germ-free NOD mice were transferred with the fecal microbiota from mice in the Ctr group; GF_KO_, germ-free NOD mice were transferred with the fecal microbiota from mice in the KO group. **(A)** Chao1, **(B)** PD whole tree and **(C)** Shannon index of gut microbiota in GF_Ctr_ and GF_KO_ mice**.** The data in **(A)-(C)** are shown as the mean ± SEM, and Student’s t-test (two-tailed) was used to analyze the GF_Ctr_ vs. GF_KO_ groups.

**Figure S7**

**Figure S7 | FITC-dextran assessment of the permeability of the gut barrier, one month after fecal microbiota transplantation**. Germ-free NOD mice were transferred with the fecal microbiota from mice in the Ctr group; GF_KO_, germ-free NOD mice were transferred with the fecal microbiota from mice in the KO group. GF_Ctr_ group, n=6; GF_KO_ group, n=6. The data are shown as the mean ± SEM, and Student’s t-test (two-tailed) was used to analyze the GF_Ctr_ vs. GF_KO_ groups.

**Figure S8**

**Figure S8 | Levels of mRNA expression of gut barrier integrity related genes in the ileum and colon tissues**. Germ-free NOD mice were transferred with the fecal microbiota from mice in the Ctr group; GF_KO_, germ-free NOD mice were transferred with the fecal microbiota from mice in the KO group. The gene expression in ileum tissue **(A)-(H)** one week or one month **(Q)-(X)** after fecal microbiota transplantation. The gene expression in colon tissue **(I)-(P)** one month after fecal microbiota transplantation. GF_Ctr_ group, n=4-5; GF_KO_ group, n=4-6. The pooled fresh fecal samples from 20-30 mice from each group were used as the microbiota source for the fecal microbiota transplantation. The pooled fecal samples better represent the collective structure of gut microbiota of that group of mice. The data are shown as the mean ± SEM, and Student’s t-test (two-tailed) was used to analyze the GF_Ctr_ vs. GF_KO_ groups.
